# Supplementary material for: Implicating the red body of Nannochloropsis in forming the recalcitrant cell wall polymer algaenan
Source: Nat Commun. 2024 Jun 27;15:5456. doi: 10.1038/s41467-024-49277-y (PMC11211512; doi:10.1038/s41467-024-49277-y)
Supplement: Supplementary file 1 — Supplementary information [file 41467_2024_49277_MOESM1_ESM.pdf]

Implicating the red body of *Nannochloropsis* in forming the recalcitrant cell wall polymer algaenan

v2.0 Protein ID	Spectrum Count	Length (aa)	MolWt (kDa)	Predicted Targeting	KOG Annotation
578349	94	267	29.1	signal peptide	Apolipoprotein D/Lipocalin
628747	65	172	18.5	signal peptide	n/a
585569	59	1166	122.0	signal anchor	n/a
606535	58	1041	108.9	signal anchor	n/a
673463	55	194	21.5	signal peptide	n/a
615027	50	354	38.1	signal peptide	n/a
611681	50	557	59.5	signal peptide	Hedgehog/Intein_dom
637672	48	1204	131.3	signal peptide	Glycosyl hydrolase, family 38 - alpha-mannosidase
637287	48	2200	237.2	signal peptide	Multidrug resistance-associated protein/mitoxantrone resistance protein, ABC superfamily
585999	44	540	60.6	signal peptide	Serine carboxypeptidases (lysosomal cathepsin A)
611700	37	373	38.8	signal peptide	n/a
598300	37	535	58.9	signal peptide	n/a
552404	34	1444	154.6	other localisation	Subtilisin kexin isozyme-1/site 1 protease, subtilase superfamily
595051	33	307	34.1	signal peptide	n/a
544243	32	441	48.2	other localisation	Translation elongation factor EF-1 alpha/Tu
608283	31	197	21.5	signal peptide	n/a
602341	30	232	26.1	signal peptide	n/a
614890	28	1139	121.7	signal anchor	n/a
595054	28	264	29.5	signal peptide	n/a
422327	27	264	28.7	signal peptide	n/a

Supplementary Table 1. Proteins with highest spectral counts in mass spectrometry proteomics of shed red bodies. Red bodies shed into the media during the course of cell division were isolated and subjected to denaturing sodium dodecyl sulfate polyacrylamide gel electrophoresis (SDS-PAGE), trypsin digestion, and proteomics mass spectrometry. The resulting peptide spectra and the predicted proteome of

Nannochloropsis oceanica were used to identify likely red body proteins. The top twenty proteins (sorted by observed number of spectra matching each protein) are shown below. Prediction of peptide targeting signal was done with HECTAR [121]

Peak frequency wavenumber (cm^{-1})	Assignment	Samples			
		**W+RB**	**W**	**RB**	**Gs W**
~3500/~3200	υ O–H of hydroxyl groups/N–H (amide A) of proteins	Y	Y	Y	Y
2970–2950/2880–2860	$υ_{as}$/$υ_s$ C–H of saturated methyl –CH_3	Y	Y	Y	Y
2935–2915/2865–2845	$υ_{as}$/$υ_s$ C–H of saturated methylene >CH_2	Y	Y	Y	Y
1750–1700	υ non-peptide carbonyl C = O				
~1743	υ C=O of acyl groups in saturated triacylglycerols	Y	Y	N	Y
~1734	υ C=O of saturated long-chain esters or ether esters	Y	N	Y	N
~1730–1720	υ C=O of saturated aldehydes*	Y	Y	N	Y
~1720–1710	υ C=O of saturated aliphatic ketones*	Y	N	Y	Y
~1730–1700	υ C=O of saturated aliphatic carboxylic acids*	Y	Y	Y	Y
1690–1610	υ C=O and υ C-N of Amide I in proteins	Y	Y	Y	Y
1600–1480	υ C–N, υ C–C and δ N–H of Amide II in proteins	Y	Y	Y	Y
1470–1370	δ C–H of CH_3, CH_2	Y	Y	Y	Y
~1413/~1236	δ C–H of α-CH_2 of the acyl chains of tri-(di or mono) glycerides/methylene and rocking in long chain CH_2.	Y	Y	N	Y
~1377 ± 20	Bending (δ, ρ, ω, τ) of OH of primary & secondary alcohols	Y	Y	Y	Y
1300–1200/1050–1000	$υ_{as}$ C–O of mixed (alkyl/aryl) ethers in carbohydrates	Y	Y	N	Y
~1244/~1171	δ C–H of CH_2 coupled with long chain C–O modes	Y	N	Y	N
1165–1155	υ of C–O–C glycosidic linkage	Y	Y	N	Y
1140–1070	$υ_{as}$ C–O of saturated unbranched ethers in carbohydrates	Y	Y	N	Y
1150–1075	$υ_{as}$ C–O of the secondary hydroxyl groups	Y	Y	Y	Y
1075–1000	$υ_{as}$ C–O of the primary hydroxyl groups	Y	Y	Y	Y
~988, ~966	υ of methylated double bond in the *cis* C–(CH_3)C=CH − CH=CH –C groups in carotene	Y	Y	Y	Y
890–810	$υ_s$ C–O of ethers/δ(C–H) tri-substituted alkene/epoxide	Y	Y	N	Y
721–718	ρ $(CH_2)_{n>4}$ of long-chain linear aliphatic chain	Y	Y	Y	Y

Supplementary Table 2. Characteristic absorption frequencies in the mid-infrared spectral range from 2000 to 650 cm^{-1} for **(W+RB)** *N. oceanica* shed cell walls and red bodies, **(W)** *N. oceanica* shed cell walls, **(RB)** *N. oceanica* shed red bodies, and **(Gs W)** *G. sculpta* shed cell walls.

υ = stretch ($υ_s$, $υ_{as}$)); $υ_s$ = symmetric stretch; $υ_{as}$ = asymmetric stretch; δ = scissoring; ρ = rocking; τ = twisting; ω = wagging.

*Band widths overlap between saturated ketones/aldehydes and saturated carboxylic acids.

References: (Baumann WJ & Ulshöfer HW, *Chem. Phys. Lipids* 2, 1968; Parkash S, Blanshard JMV, *Spectrochimica Acta*, 31A, 951-57, 1975; Pond JL et al. *Science*, 231:1134-1136, 1986; Skkal M et al. *Mikrochim. Acta*, 112:1-10, 1993; Rodriguez MC et al. *J. Phycol*, 35: 1025-1031, 1999; Smith BC, *Infrared spectral interpretation: A systematic approach*, CRC press LLC, 265pp, 1999; Salmon E et al. *Organic Geochemistry*, 40: 400-415, 2009; Lewise RNAH and McElhaney RN, *Biochimica et Biophysica*

Acta 2012; Miglio R et al. *J. Appl Phycol*, 25:1621-1631, 2013; Scholz MJ et al. *Eukaryotic Cell*, 13(11):1450-1464, 2014); J Coates, Interpretation of IR spectra, a practical approach.

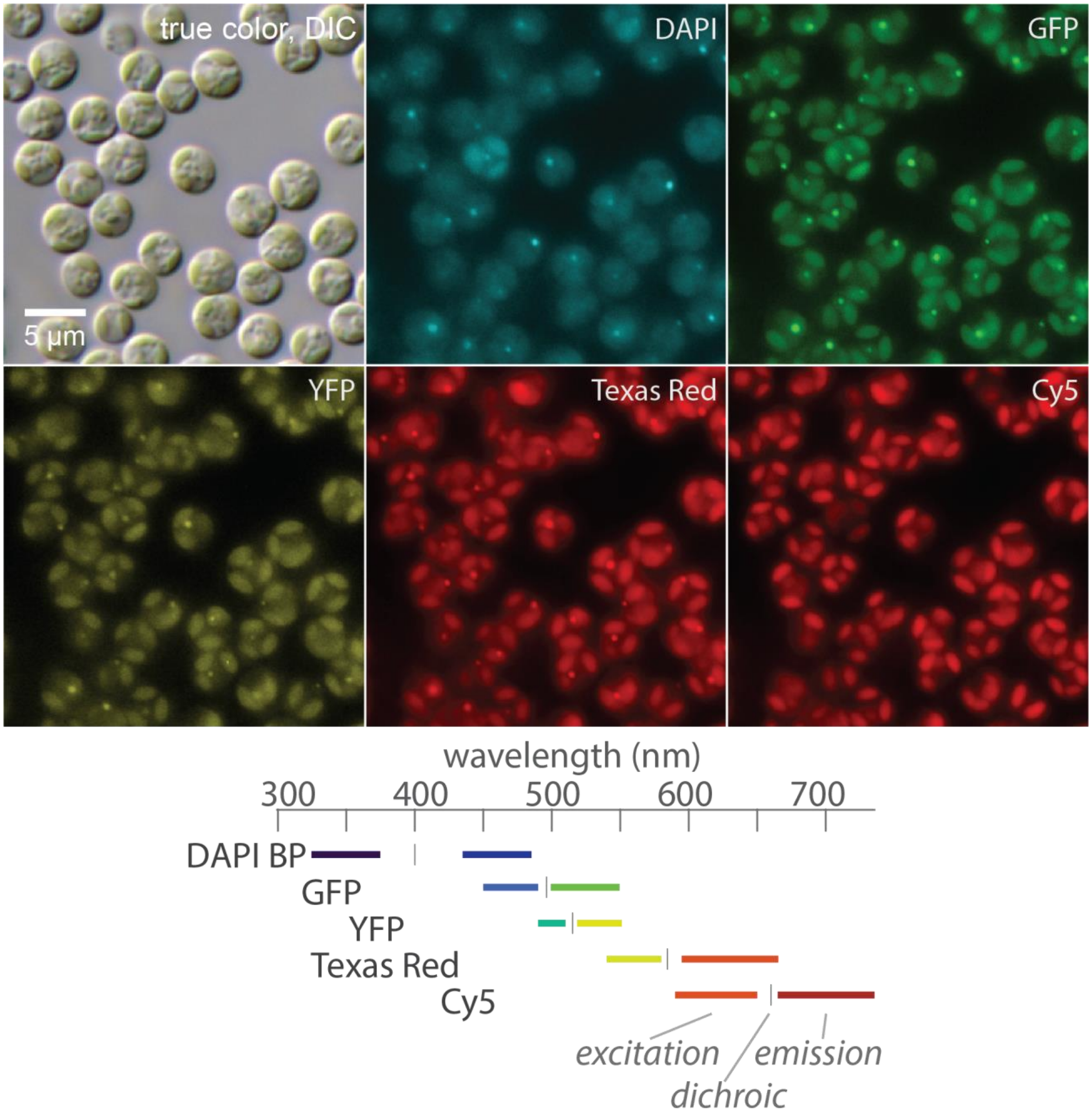

Supplementary Figure 1. Apparent red body autofluorescence through different filter sets seen. Pertinent to Figure 1. Synchronously dividing cells grown in a 12-h light/12-h dark photoperiod were imaged on a wide-field fluorescence microscope. For fluorescence images, shown are 2D maximum intensity projections from three z positions that spanned the majority of the cell volumes. Channel labels refer to the filter sets used; all signal is derived from autofluorescence and false colored to approximate true color (except for DAPI, displayed in cyan for visibility). Filter set specifications are graphically depicted at the bottom. Scale bar = 5 µm and applies to all images in this figure.

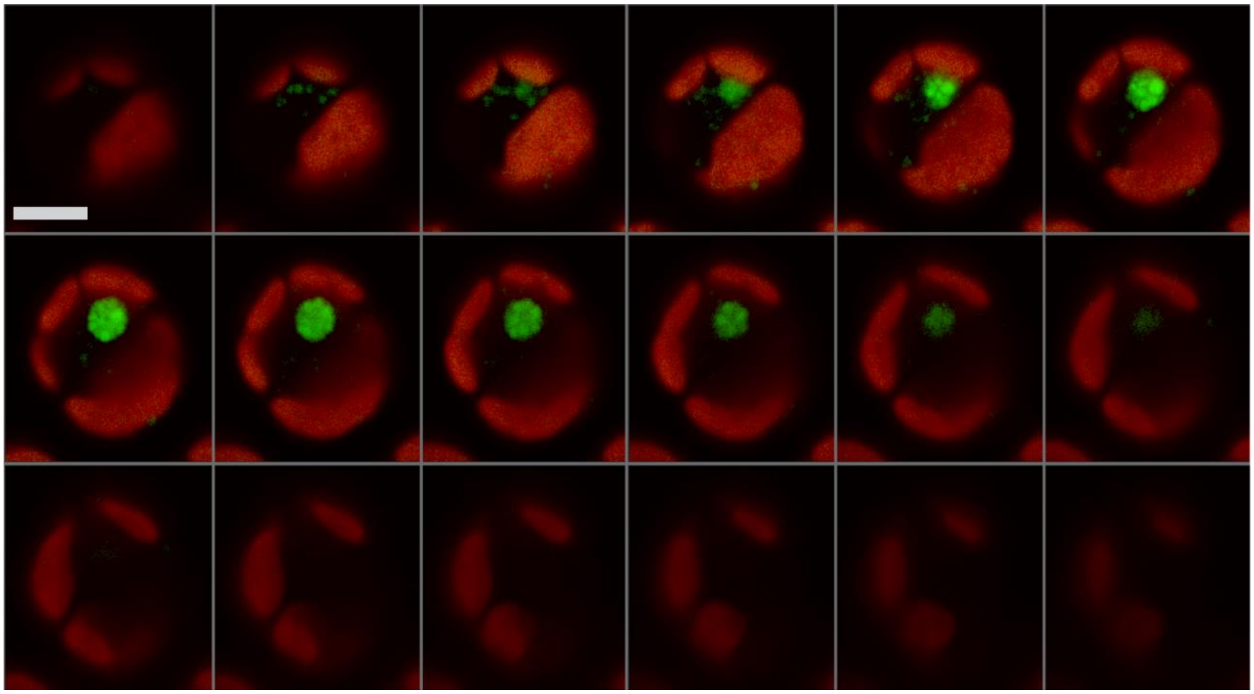

Supplementary Figure 2. Some eustigmatophyte red bodies appear to be aggregations of smaller globules. Pertinent to Figure 1. Confocal scanning laser microscopy z-stack of a single *Vischeria* sp. cell. Green autofluorescence (pseudo colored green, ex/em: 488 nm / 525 nm), and chlorophyll autofluorescence (pseudo colored red, ex/em: 633 nm / >680 nm). Each slice shown was collected slightly less than 0.5 μm apart in the z dimension. Scale bar = 5 μm and applies to all images in this figure.

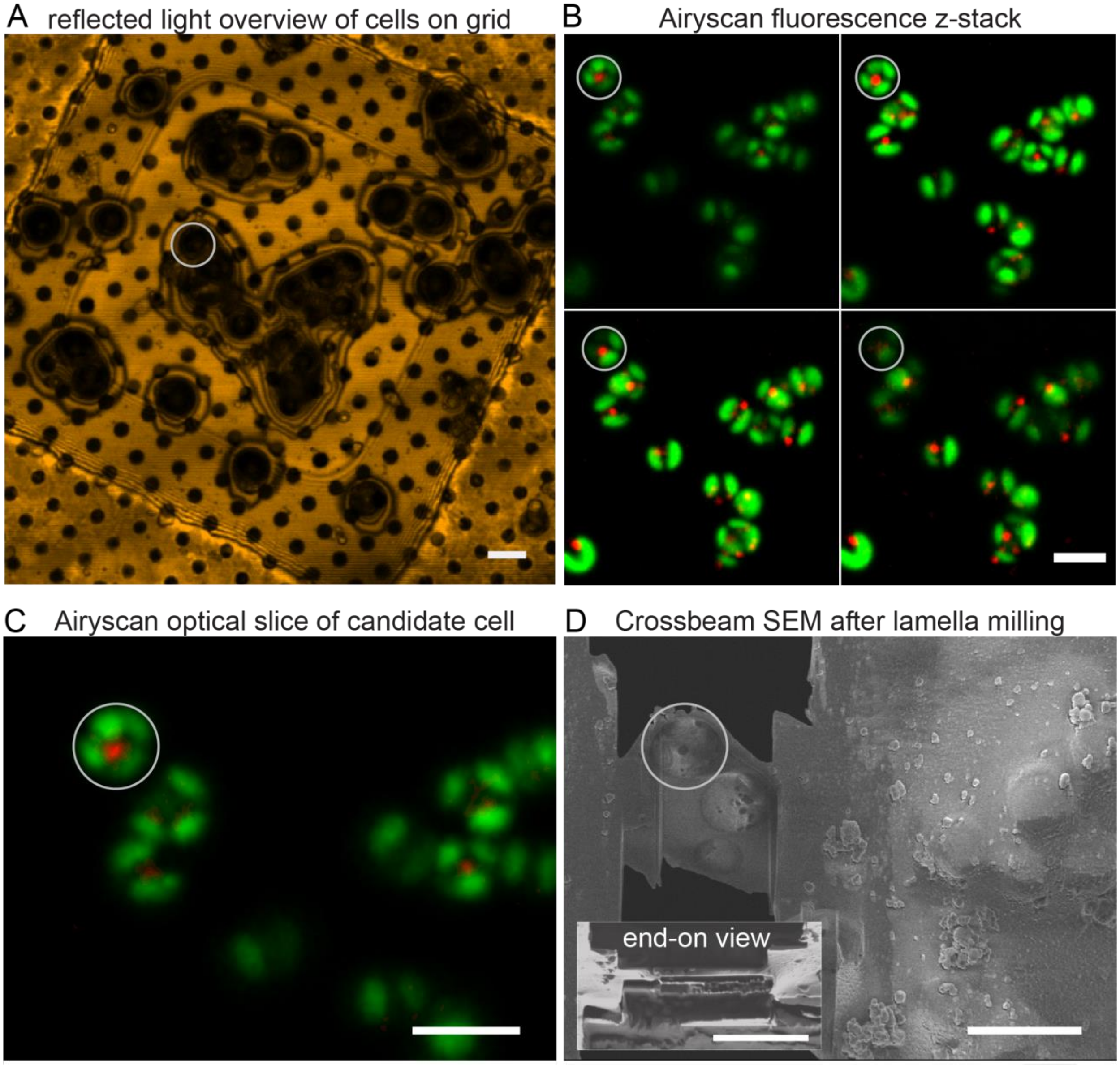

Supplementary Figure 3. Lamellae preparation for cryo electron tomography was guided by correlative light microscopy. Pertinent to Figure 2. **(A)** A relatively low magnification "overview" image was acquired by reflected light of frozen cells on the carbon grid. **(B)** Cryo autofluorescence z-stacks were acquired to locate candidate red bodies (red false colored) among chloroplasts (green false colored) in three dimensions. A subsample of a z-stack is shown here for brevity. Note that the red/green false colors were rendered differently here than in other images in this manuscript. **(C)** Optical section of a candidate cell that contained a prominent red body (red false colored) and distinct chloroplast autofluorescence features to guide FIB-SEM, and identify features in cryo-TEM images. **(D)** SEM views of a finished physical section (lamella) after beam milling of the candidate cell in preparation for cryo TEM tomography. The main panel shows the lamella "top down"; inset shows an example lamella "end-on". The scale bar = 5 μm in all images. The same candidate cell is circled in each image throughout the process.

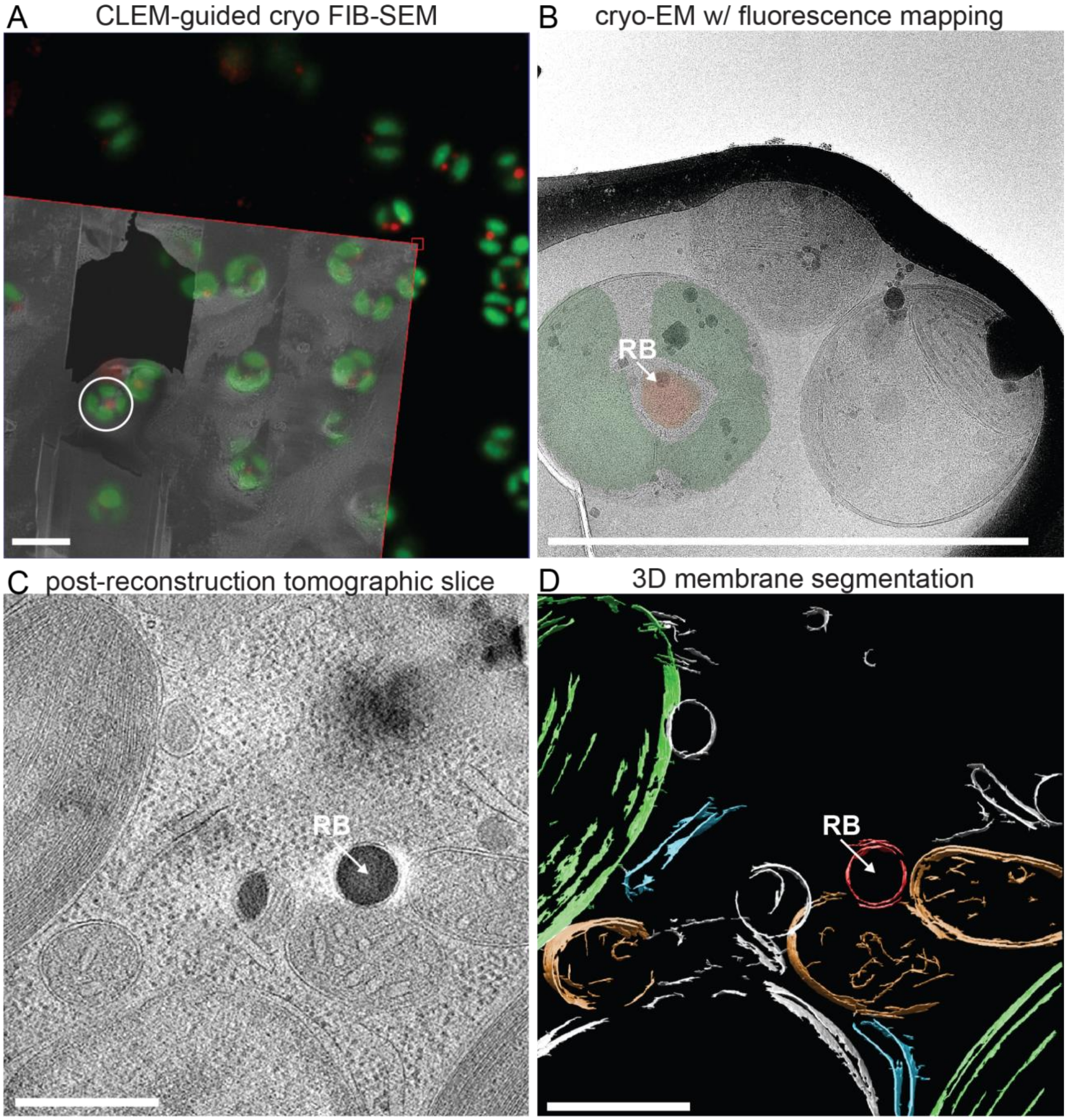

Supplementary Figure 4. Cryo electron tomography pipeline

Pertinent to Figure 2. **(A)** Putative red bodies were targeted for cryo-FIB milling using CLEM. 4 to 5 areas per specimen were selected for lamellae milling and cryoET data collection. **(B)** Low magnification overview of the same section in cryo EM before collecting tilt series. The fluorescence from CLEM grid mapping is overlaid on the section to confirm the position of the putative red body in the selected cell (circled in A). **(C)** A slice from the final tomographic volume after 3D reconstruction, highlighting the targeted red body in its cellular context. A video through the entire 350 nm thick tomographic volume is available as Video 1. **(D)** View of a 120 nm slice through the 3D segmented cellular compartments of the same tomogram using the TomosegmemTV membrane detection software. The red body boundaries are labeled in red, chloroplasts in green, mitochondria in orange and endoplasmic reticulum in cyan. Scale bars are 5 µm in (A) and (B), 500 nm in (C) and (D). Putative red body indicated by white “RB” and arrow in (B), (C), and (D).

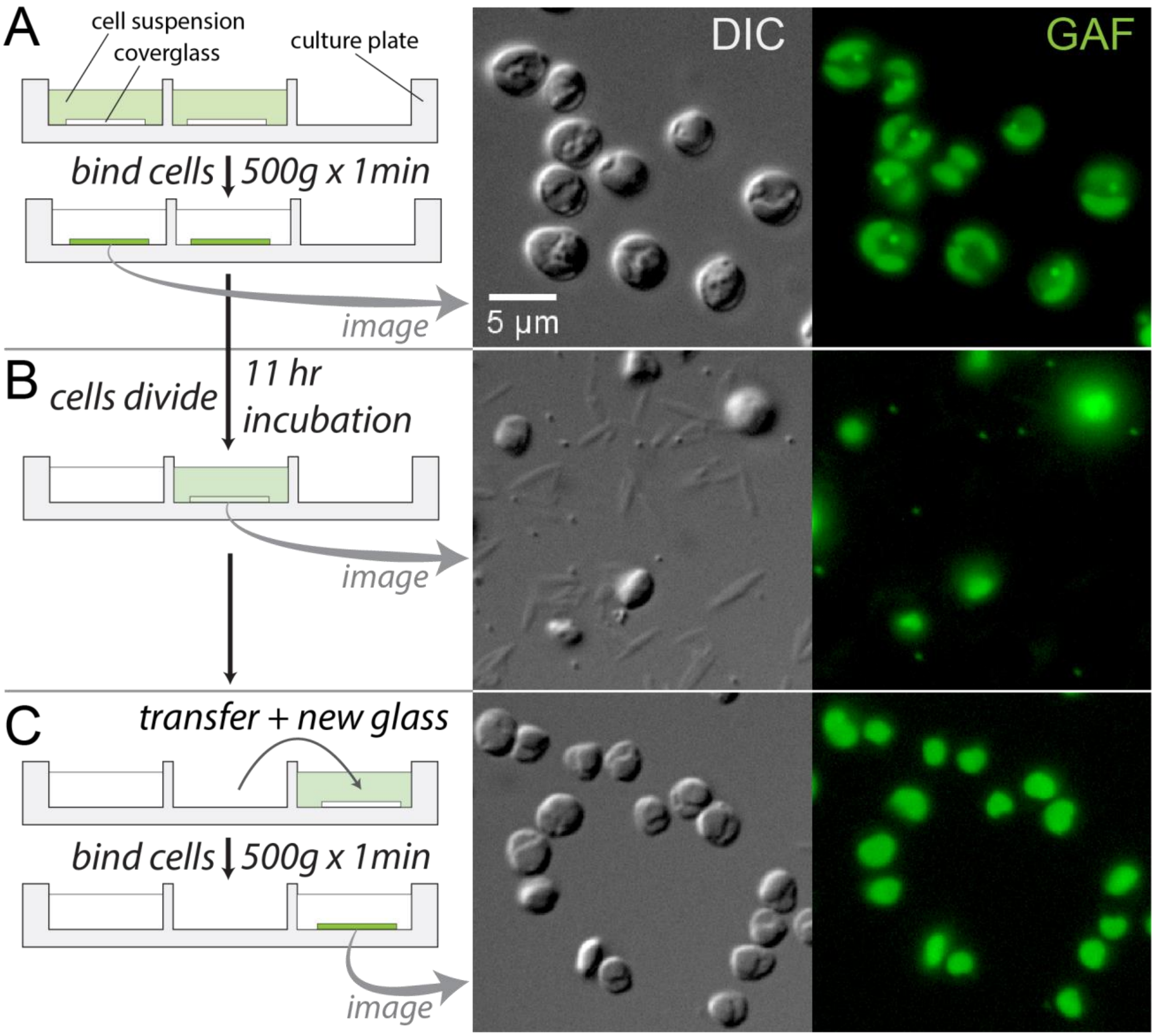

Supplementary Figure 5. The red body is shed with the autosporangial wall upon autospore release. Subpanel labels A, B, and C refer to horizontal groupings consisting of a cross section schematic of the experimental procedure involving a 6 well culture plate, and accompanying micrographs to the right. **(A)** Aliquots of a synchronously dividing liquid culture entrained to a 12 hour light, 12 hour dark photoperiod were bound at subjective dusk to polylysine-coated microscope coverslips by centrifugation in cell culture plate wells. The first coverslip was imaged immediately for DIC transmitted light and green autofluorescence (GAF, excitation 488 nm, emission 495 to 550 nm). The cell culture plate was placed in the dark with gentle shaking. **(B)** 11 hours after subjective dusk, cells had divided, and the second coverglass was imaged. The media had regained a green color, presumably from released autospores no longer bound to the coverglass. Putative shed red bodies and autosporangial walls remained affixed. **(C)** The green colored media was transferred to a new well, and cells re-bound onto a new coverglass and imaged. These recently divided cells are smaller and have not yet developed visible red bodies. The scale bar = 5 µm in all images. This experiment was performed at least 5 times with slight variations in procedure yielding similar results.

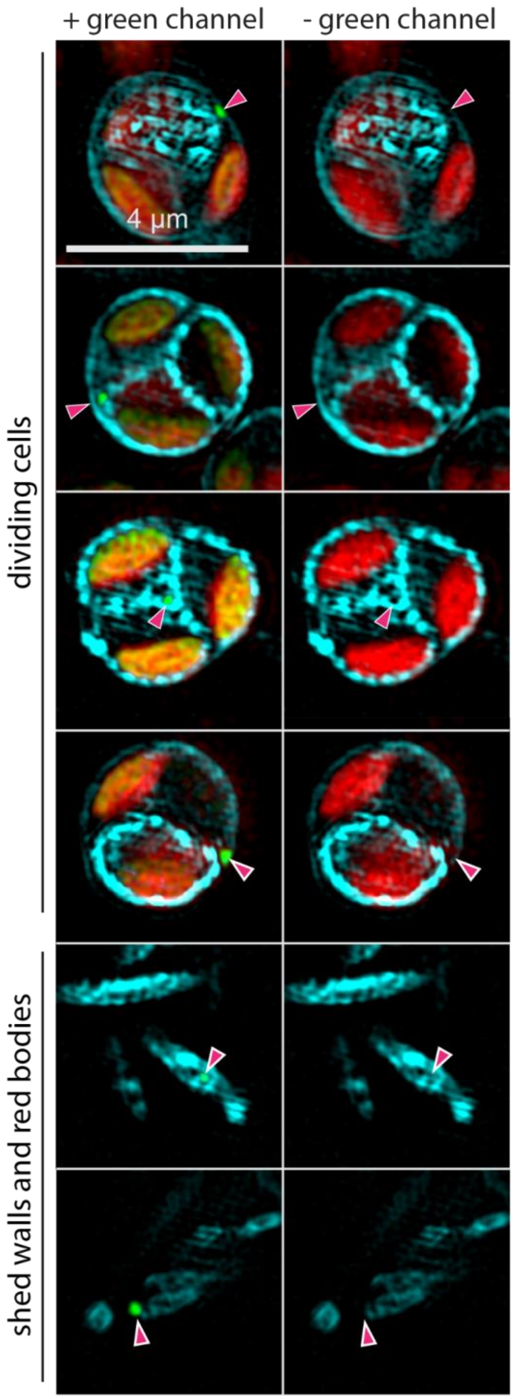

Supplementary Figure 6. The red body resides in the apoplast during autospore cell wall formation. Pertinent to Figure 4. Synchronously dividing cells were sampled 3 hours after subjective dusk and bound to polylysine-coated coverslips. These were incubated for 1 hour in the dark with media + 0.05% calcofluor white, a cellulose-binding fluorescent dye. These cells were imaged with SIM (cyan = excitation 405 nm, emission 420-480 nm; green = excitation 488 nm, emission 495-550 nm; red = excitation 642 nm, emission >655 nm). Displayed are example cells that exhibited visibly stained cell walls, each with and without the green channel to allow examination of the area around the green fluorescence of the red body (indicated by magenta arrowhead). In some cases, what appeared to be shed autosporangial walls were visible with fluorescent punctae visible within (lower two image

pairs). Scale bar = 4 microns and applies to all images. This experiment was performed at least 5 times with slight variations in procedure yielding similar results.

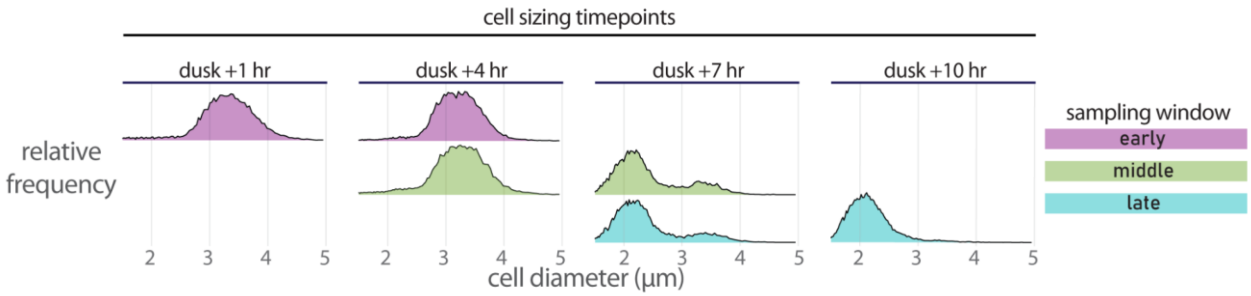

Supplementary Figure 7. Cell sizing at beginning and end of staining windows confirms synchronous autospore release. Pertinent to Figure 4. Cell diameter was quantified with a Coulter counter at the indicated time points. Each window was sampled at the beginning and end, so the dusk +4 h and dusk +7 h time points show cell size distributions for two samplings, which can detect perturbations in division due to culture handling. Distributions are plotted as relative frequency on the y axis, with the same vertical scaling (bin size ~ 0.01 μm, maximum vertical height ~3.6%, for each distribution, n > 10k). Results are shown for one experiment out of the three performed.

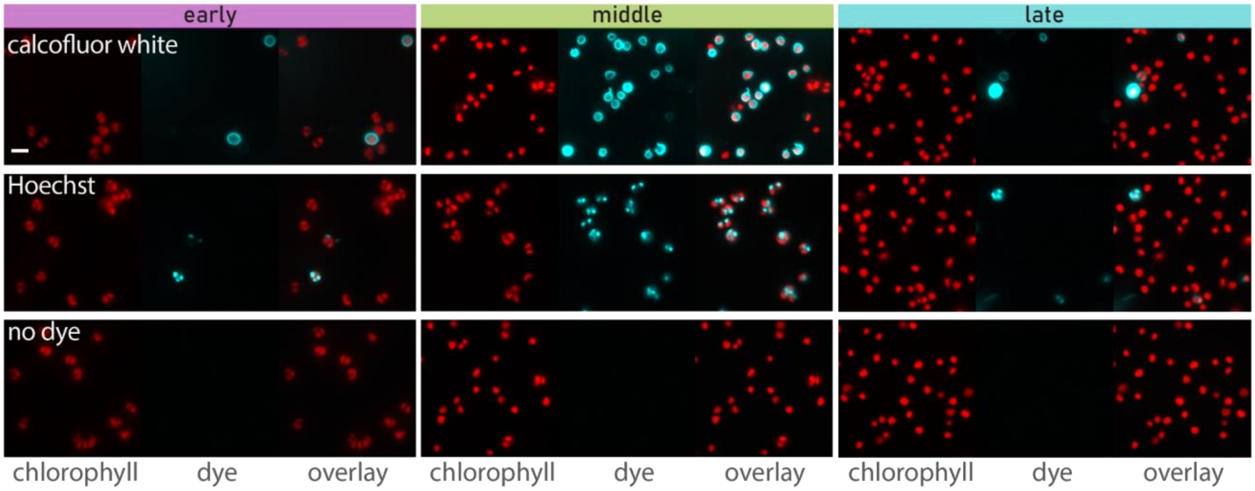

Supplementary Figure 8. Additional microscopy of CFW and Hoechst stained cells. Pertinent to Figure 4. Images were acquired and are presented in a similar way as in Figure 5. Images are of cells from a different independent experiment from those presented in Figure 5 (n=3 biologically separate trials with 7 technical replicates for each stain/timepoint combination.). The scale bar in the upper left image = 5 μm, and applies to all images.

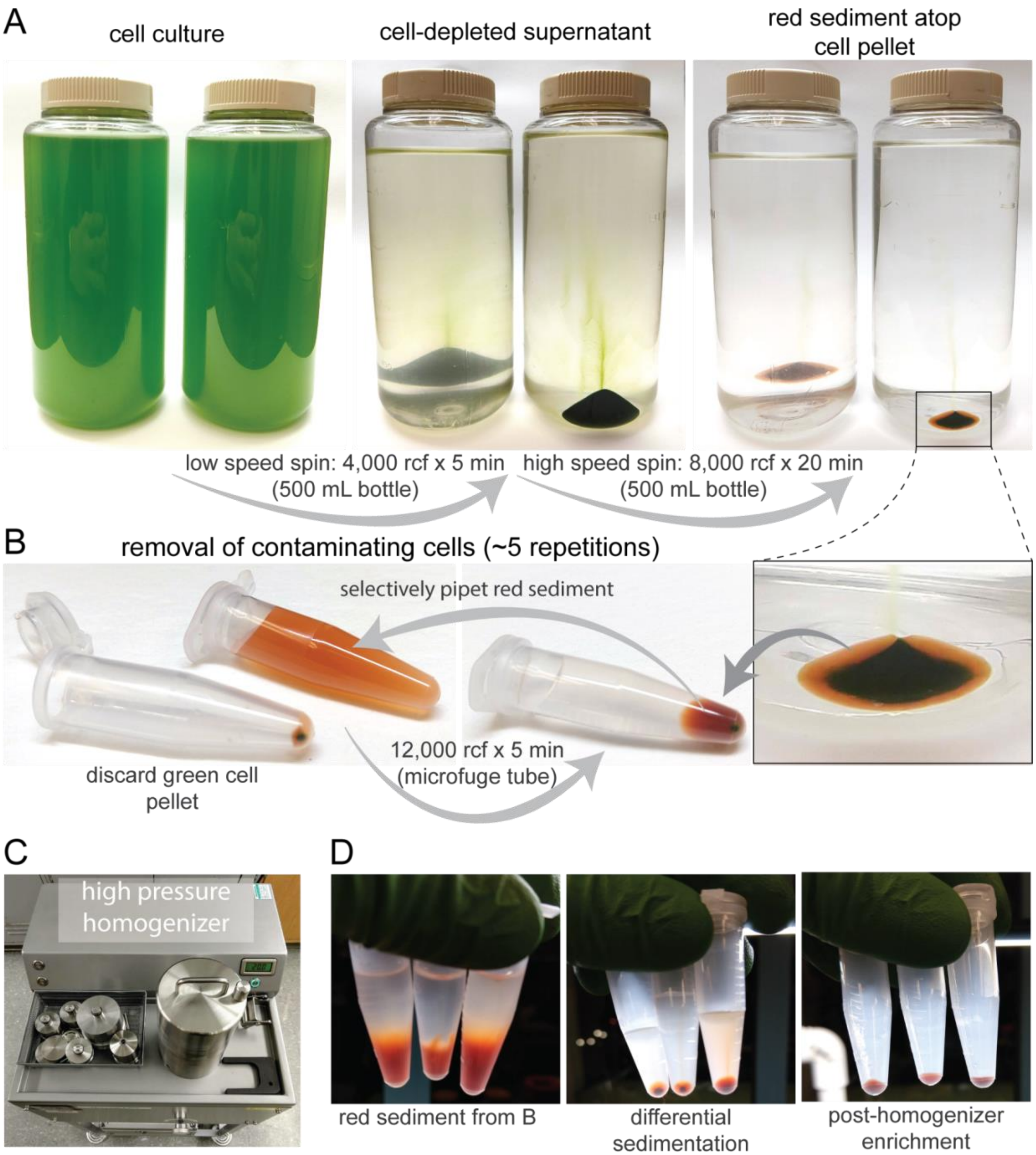

Supplementary Figure 9. Isolation procedure of the red sediment and isolation of shed red body particles. Pertinent to Figure 6. **(A)** A procedure to isolate shed walls and red bodies from high density liter scale liquid cultures grown at 3% CO_2 and 100 μmol photons m^{-2} s^{-1}. A relatively low intensity centrifugation first depletes the supernatant of whole cells, and this supernatant is then subjected to a higher intensity centrifugation to pellet remaining cells and red sediment. **(B)** The pellet from (A) is further depleted of cells by repeated centrifugation in microfuge tubes and selective pipetting to avoid transferring the lower cell pellet to the next repetition. **(C)** The isolated red sediment from (B) is passed through a high-pressure homogenizer and recollected in microfuge tubes. **(D)** Further differential sedimentation to isolate the shed red body particles. Left panel- original red sediment as in (B), middle panel- samples after homogenizer treatment and the first round of differential sedimentation as in (B), except the dark lower pellet is retained for subsequent repetitions. Right panel- the dark pellet after several rounds of depletion of residual shed wall particles.

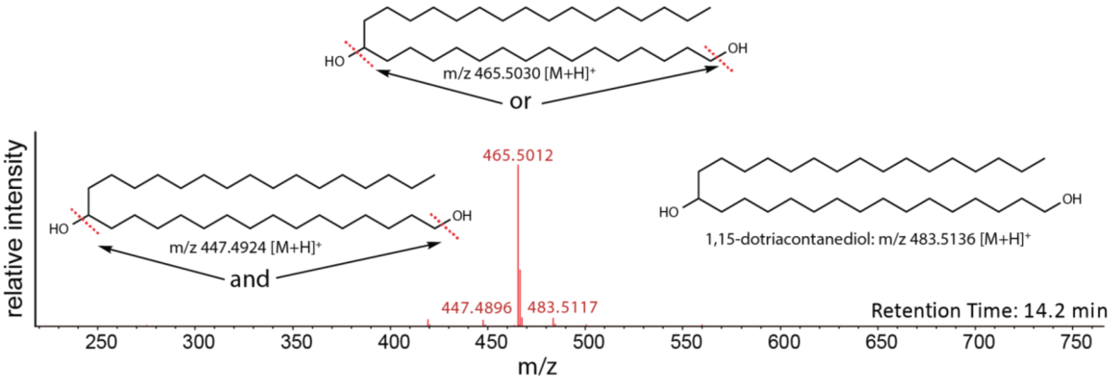

Supplementary Figure 10. APCI In-source fragmentation of putative C_{32} long-chain diol. Pertinent to Figure 6. C_{32} long-chain diol is depicted as 1,15-dotriacontanediol based on long chain diols characterized in Balzano et al. (2019). m/z values measured are within 5 ppm of predicted m/z for the identified ions.

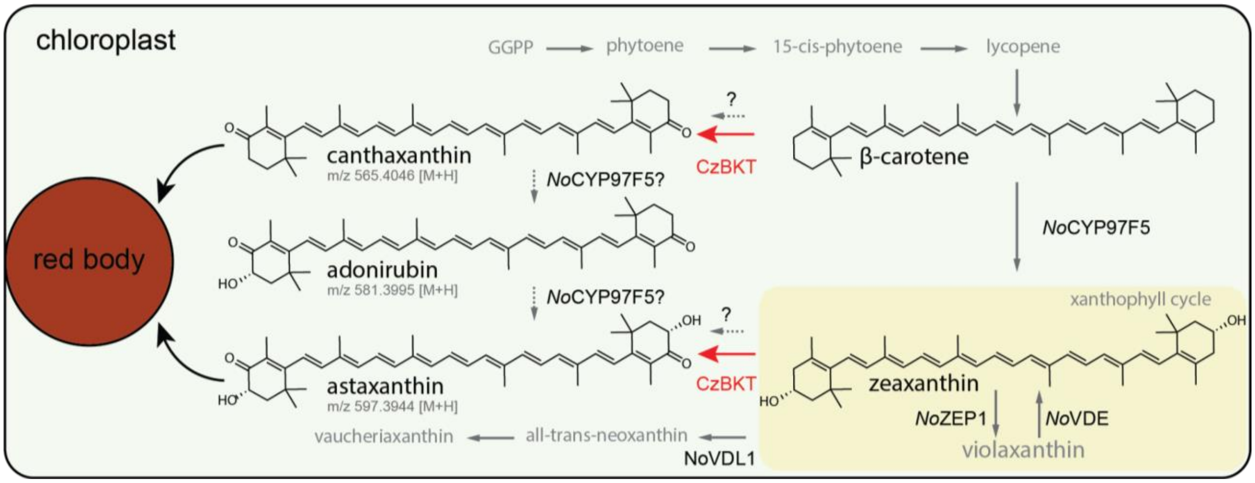

Supplementary Figure 11. Rationale for *CzBKT* overexpression. Pertinent to Figure 7. A schematic biosynthetic pathway for carotenoids in *Nannochloropsis* is shown. While the endogenous pathway leading to canthaxanthin and astaxanthin are not precisely known (dotted lines). NoCYP97F5 have previously been shown to catalyze C3 & C3' hydroxylation of beta-carotene [134], similar to the hydroxylation required for astaxanthin biosynthesis from canthaxanthin. Heterologous expression of *C. zofengensis* beta-carotene ketolase (*Cz*BKT) led to ketocarotenoid accumulation and downstream effects on the red body size and its carotenoid composition.

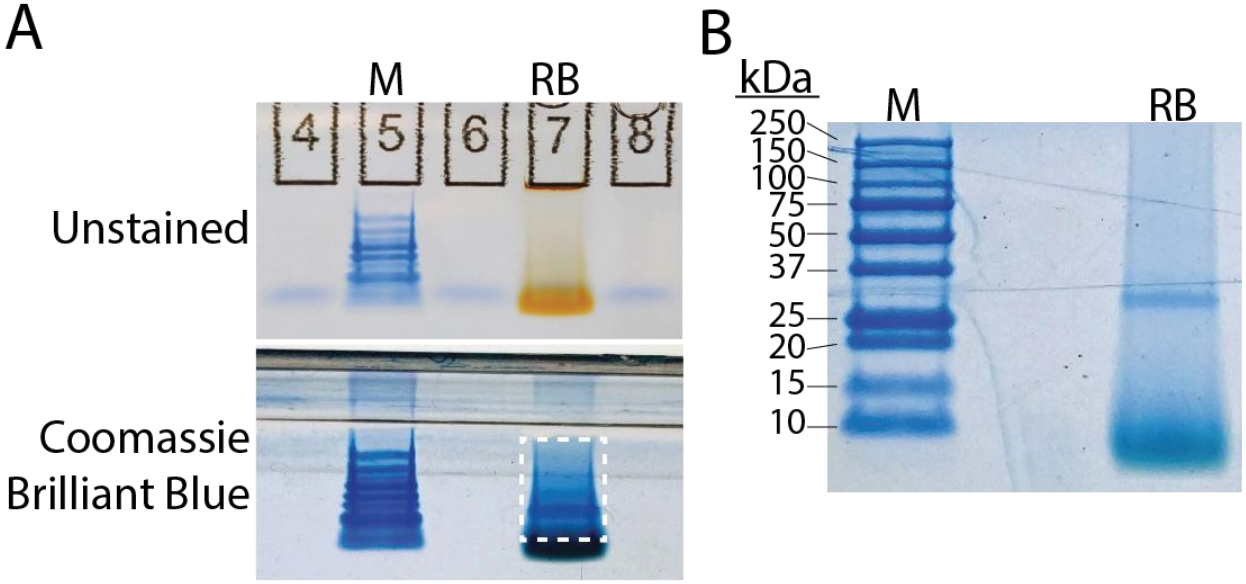

Supplementary Figure 12. SDS-PAGE preparation of protein extracts from shed red bodies for proteomics mass spectrometry. Pertinent to Supplementary Table 1. **(A)** “Red sediment” mixture of shed cell walls and red bodies was subjected to high pressure homogenization and differential centrifugation to enrich for red bodies (RB). Isolated red bodies were solubilized in denaturing sodium dodecyl sulfate (SDS) protein extraction buffer and subjected to polyacrylamide gel electrophoresis (SDS-PAGE). M = protein marker ladder. Upper image is of the unstained gel, showing the naturally occurring red orange pigmentation of the extract. Every other lane was loaded with SDS extraction buffer blanks for homogeneity across the gel, and the loading buffer band can be seen at the bottom of the gel. The lower image shows the same gel after Coomassie Brilliant Blue staining. The white dotted line indicates the approximate region manually excised and used for subsequent proteomics. **(B)** A sample similar to the one shown in (A) was run, but for a longer duration to visualize potential bands that would denote possible abundant proteins, e.g. the clear band between 25 and 37 kilodaltons (kDa).

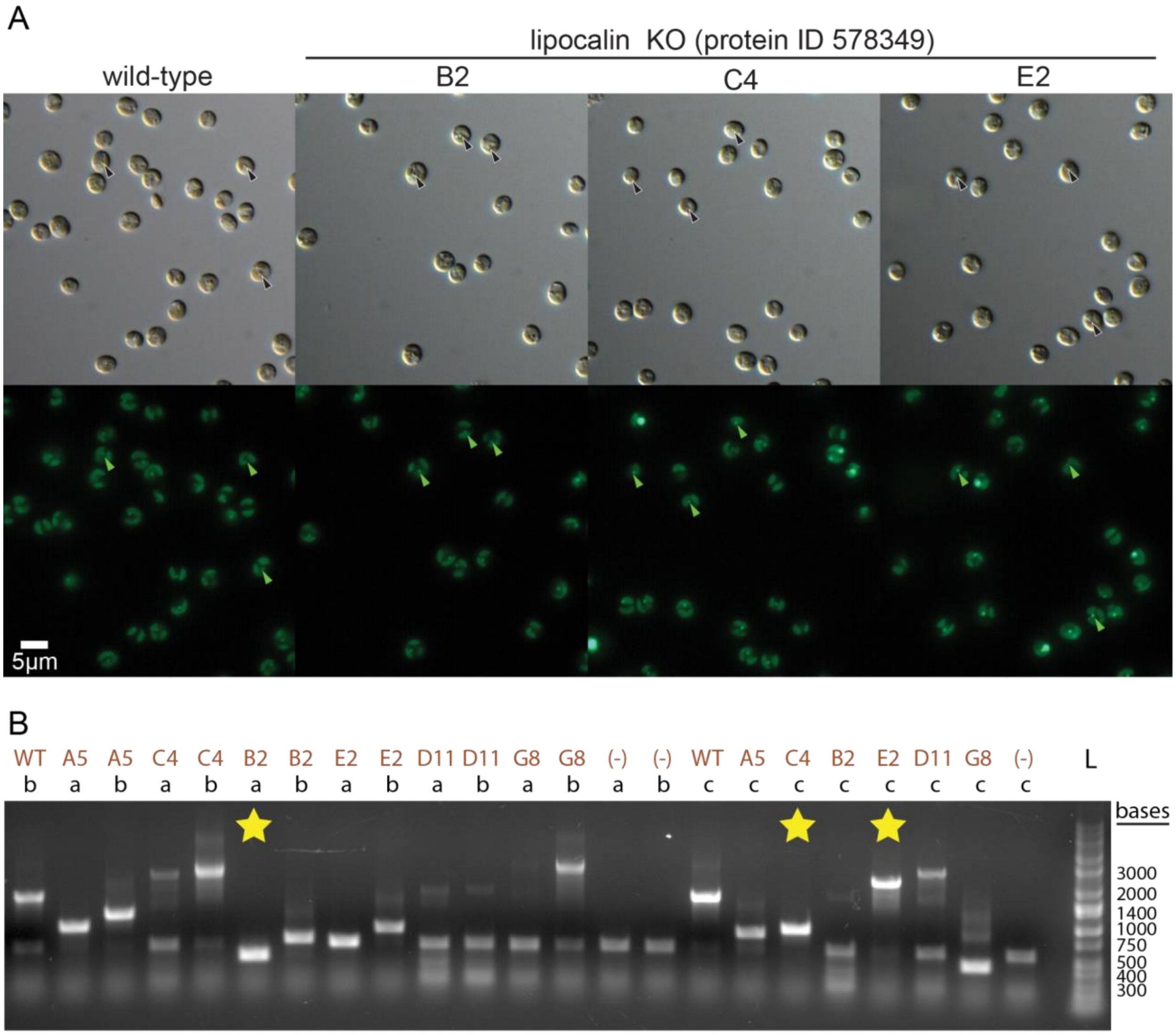

Supplementary Figure 13. CRISPR-Cas9 mediated knock-out mutants of a candidate red body protein do not show an obvious defect in red body autofluorescence or cellular morphology. Pertinent to Supplementary Table 1. **(A)** Three independent strains (designated B2, C4, E2) of putative knock out mutants of the protein ID 578349 "Apolipoprotein/lipocalin" were examined by differential interference contrast light microscopy (DIC, top row of images) and green autofluorescence (lower row of images). Arrowheads indicate example red bodies in both DIC and green autofluorescence, which were present in both wild type and the mutants. **(B)** Genotyping agarose electrophoresis gel showing PCR products amplified from the expected mutation sites in the genome for gene 578349. Expected amplicon from wild type (WT) = 1458 bp. Expected amplicon size from a full deletion between the two gRNA binding sites used = ~400 bp. Yellow stars indicate strains selected for the microscopy presented in (A), which had a single, bright band clearly of a different size than the WT, indicating a large deletion (B2 and C4) or an insertion (E2).

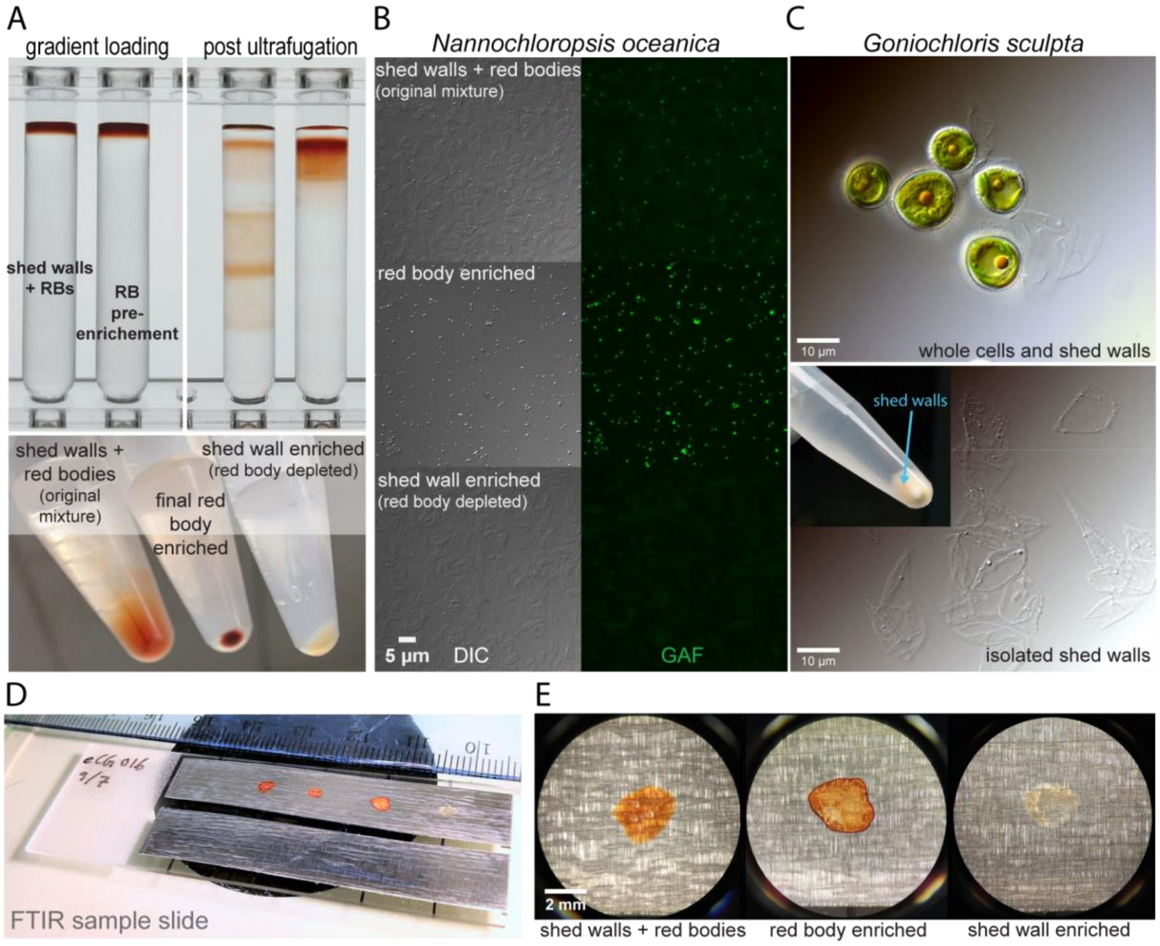

Supplementary Figure 14. Preparation of FTIR samples.
Pertinent to Figure 8. **(A)** Discontinuous sucrose density gradient to separate residual shed wall particles from the red body enrichment. Left panel- initial loading of the centrifuge tubes at the top of 20%, 26.7%, 33.3% and 40% sucrose (w/v with water). Left tube was loaded with the red sediment mixture of shed walls and red bodies, right tube with the high pressure homogenizer treated red sediment as described in Figure 7- supplemental 1. Right panel- after 30 minutes at 40,000 rcf. The final enrichment consisted of the dark band at the very top of the right tube. Bottom panel- one of the water wash steps (of 5 total) after recollection from the gradient, plus a red body depleted sample of the original mixture incubated with 1% sodium dodecyl sulfate (SDS) at 50°C for 10 min. **(B)** Quality control microscopy of the enriched fractions. Samples from (A) were bound to coverglasses and imaged as described previously. GAF = green autofluorescence. Scale bar = 5 microns and applies to all images in panel B. **(C)** Isolation of apparent shed walls from *G. sculpta* cultures. Upper panel- cells and shed walls from the agar culture plate. Lower panel- shed walls after differential sedimentation and selective pipetting (see inset). (**D)** Samples dried down onto aluminum foil in preparation for FTIR measurement. **(E)** View of dried samples through dissecting scope ocular. Three separate images joined to present them side by side.

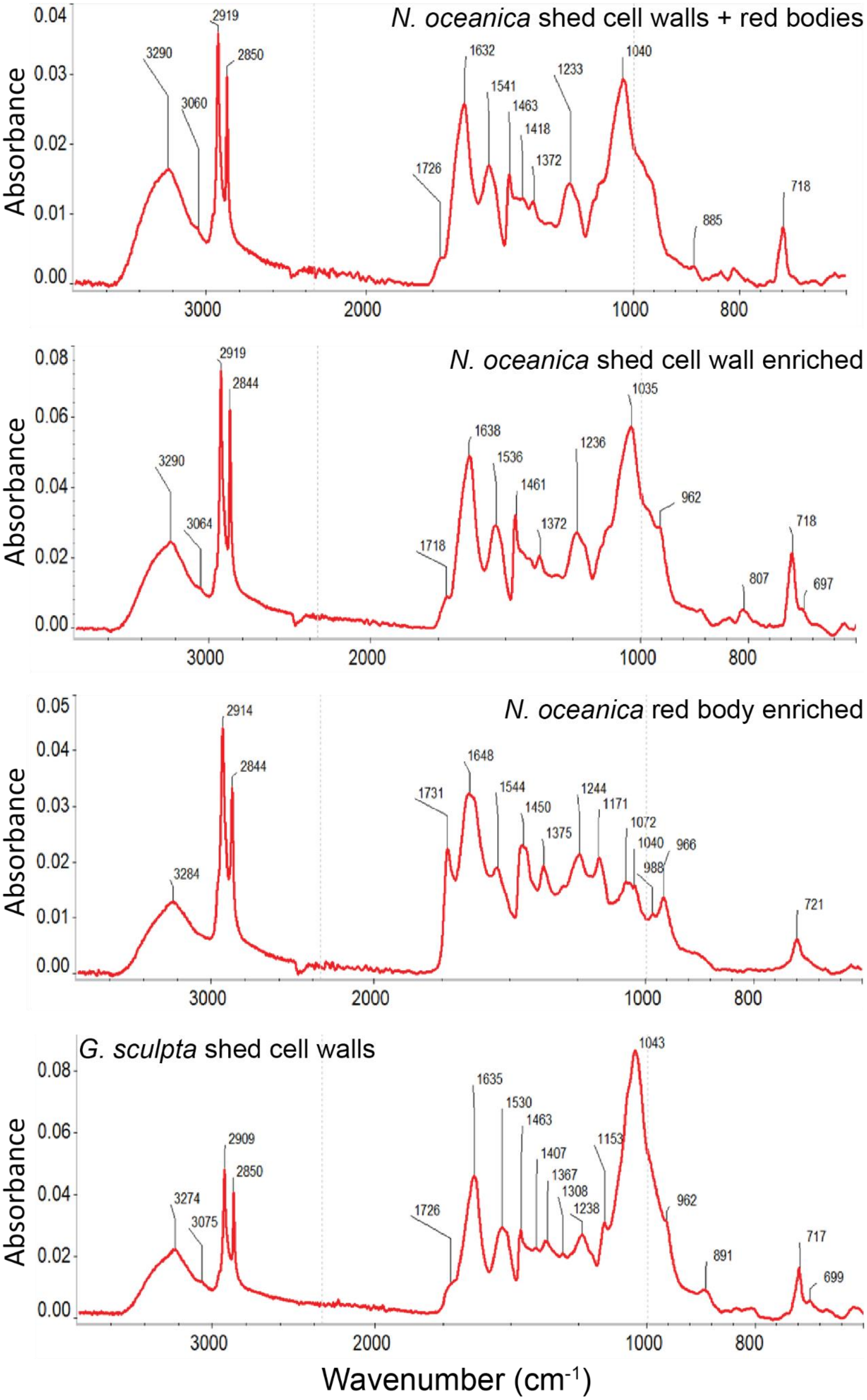
N. oceanica shed cell walls + red bodies
3290
3060
2919
2850
1726
1632
1541
1463
1418
1372
1233
1040
885
718
N. oceanica shed cell wall enriched
3290
3064
2919
2844
1718
1638
1536
1461
1372
1236
1035
962
807
718
697
N. oceanica red body enriched
3284
2914
2844
1731
1648
1544
1450
1375
1244
1171
1072
1040
988
966
721
G. sculpta shed cell walls
3274
3075
2909
2850
1726
1635
1530
1463
1407
1367
1308
1238
1153
1043
962
891
717
699
Absorbance
0.00
0.02
0.04
0.06
0.08
3000
2000
1000
800
Wavenumber (cm-1)

Supplementary Figure 15. ATR-FTIR absorbance spectrum peak calls. Pertinent to Figure 8. Infrared absorbance spectra from Figure 8 are shown with local maxima annotations (in wavenumber). Additionally, included here is a spectrum for the shed cell walls of the eustigmatophyte alga, *Goniochloris sculpta*.

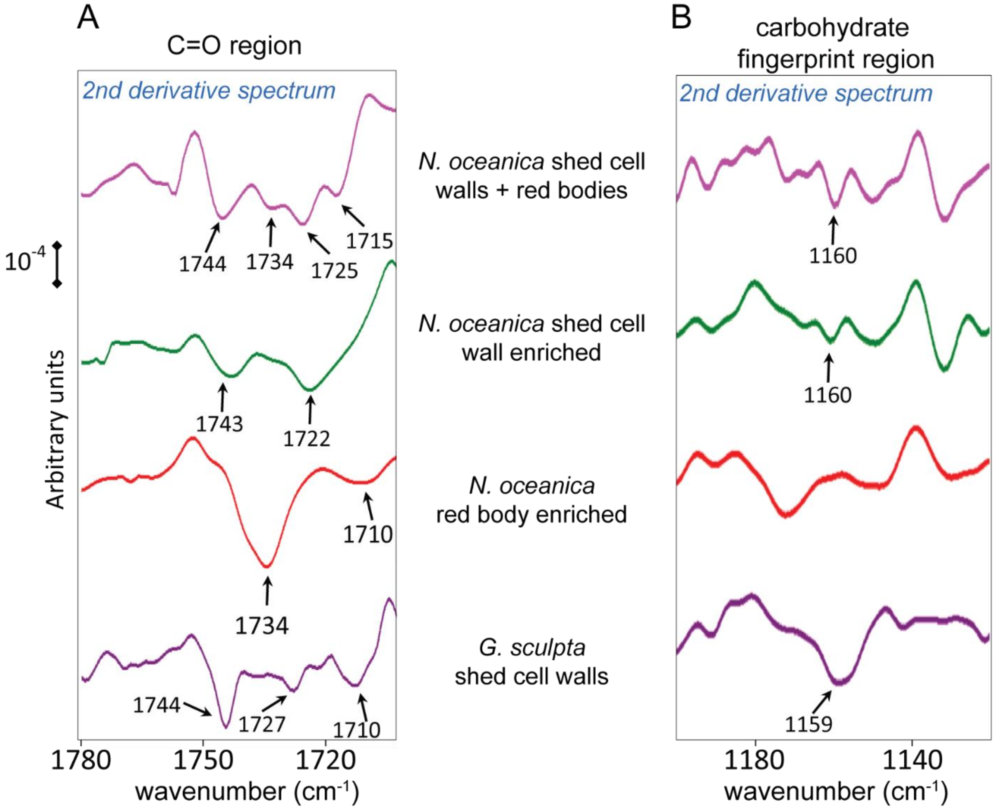

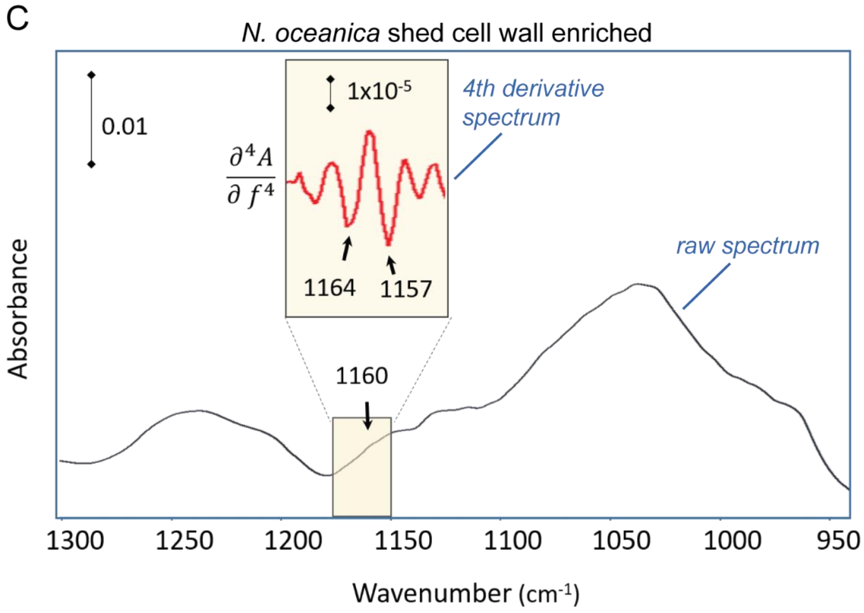

Supplementary Figure 16. Analyses of second and higher order derivatives of ATR-FTIR spectra reveal additional information about chemical composition. Pertinent to Figure 8. The prominent bands in the raw absorption spectra if Figure 8 were identified by the corresponding second derivative spectra.

(A) Second derivative spectra and peak calls for the non-peptide carbonyl region, and **(B)** a segment of the carbohydrate fingerprint region. **(C)** The shoulder at 1160 cm^{-1} was further decomposed by 4th derivative analysis.

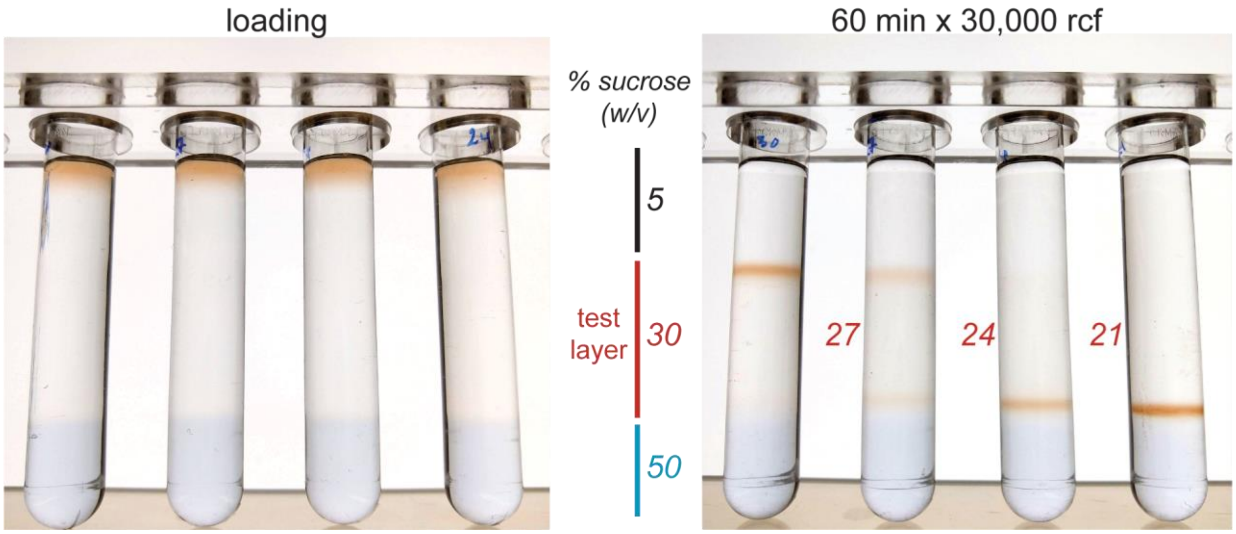

Supplementary Figure 17. Density estimation of isolated shed red bodies by discontinuous sucrose gradients. Pertinent to Figure 8. Shed red bodies were isolated as shown in Figure 9- supplemental 1, and loaded onto sucrose step gradients composed of a 5% (w/v) focusing layer, a test layer (either 30%, 27%, 24%, or 21%), and a stopping layer (50%) made visible with a small amount of bromophenol blue. Gradients were subjected to 30,000 relative centrifugal force (rcf) for 60 min at 20°C, and examined to see if the red bodies sedimented above or below the test layer.
