# Supplementary material for: Implicating the red body of Nannochloropsis in forming the recalcitrant cell wall polymer algaenan
Source: Nat Commun. 2024 Jun 27;15:5456. doi: 10.1038/s41467-024-49277-y (PMC11211512; doi:10.1038/s41467-024-49277-y)
Supplement: Supplementary file 3 — Description of Additional Supplementary Files [file 41467_2024_49277_MOESM3_ESM.pdf]

Description of Additional Supplementary Files

Supplementary Data Legends:

Supplementary Data 1. Spreadsheet containing the oligos used in cloning constructs and genotyping for CzBKTox mutants. This spreadsheet lists the oligo sequences, destination plasmids, and cloning method used to create CzBKTox transformation constructs. Additionally, oligos used to sequence the NoLCN mutant strains.

Supplementary Data 2. Raw UHPLC-HRMS data from WT cells. Raw data gathered from whole cell extractions of WT Nannochloropsis oceanica cells in .mzML format.

Supplementary Data 3. Raw UHPLC-HRMS data from CzBKTox mutant cells. Raw data gathered from whole cell extractions of CzBKTox mutant Nannochloropsis oceanica cells in .mzML format.

Supplementary Data 4. Raw UHPLC-HRMS data from red sediment samples. Raw data gathered from red sediment extractions of WTt Nannochloropsis oceanica cells in .mzML format.

Supplementary Data 5. Raw UHPLC-HRMS data from red body enriched samples. Raw data gathered from whole red body enriched extractions of CzBKTox mutant Nannochloropsis oceanica cells in .mzML format.

Supplementary Movie 1. Cryo electron tomography of a Nannochloropsis oceanica cell.
Cryo-TEM tilt series reconstruction of a 350 nm thick lamella of Nannochloropsis oceanica. The video thr ough sequential sections of the entire reconstructed cryo-tomographic volume (spaced by 4.42 nm) thro ugh the entire reconstruction shows the cellular context of a complete red body volume (spherical, elect ron-dense feature in center-right of frame). Scale bar = 500 nm. Presumptive assignments for organelles are annotated in the video (C = chloroplast, N= nucleus, M = mitochondrion, RB = red body). A still fram e of this video appears in Figure 2, as well as Supplementary Figure 4. The entire 3D cryo-tomogram as well as tilt-series data are available in the EMDB archive (accession code EMD-43893[https://www.ebi.a c.uk/emdb/EMD-43893]).
